# DOES RECEIVING SUPPORT HURT? PAIN AND SLEEP IN OLDER ADULTS’ EVERYDAY LIVES

**DOI:** 10.1093/geroni/igz038.2865

**Published:** 2019-11-08

**Authors:** Meng Huo, Jamie l Fuentecilla, kate Leger, Karen l Fingerman

**Affiliations:** 1 The University of Texas at Austin, Austin, Texas, United States; 2 university of kentucky, Lexington, Kentucky, United States

## Abstract

Pain is prevalent in late life and may cast negative impacts on older adults’ sleep. We examined this link in older adults’ everyday lives and asked whether receiving support on days when older adults had pain improved or worsened their sleep. We drew on the Daily Experiences and Well-being Study; over 300 adults aged 65+ reported on their pain, sleep and social support received throughout each day across 5 days. Multilevel models revealed that older adults in greater pain were more likely to nap throughout the day and to incur sleep disturbances at night. Older adults who slept better at night reported less pain the next day. The link between pain and sleep disturbances was stronger on days when older adults received support compared to days when they did not. This study adds to the literature regarding pain and sleep and explores what roles social factors play in this link.

